# ACSL4 is essential for radiation-induced intestinal injury by initiating ferroptosis

**DOI:** 10.1038/s41420-022-01127-w

**Published:** 2022-07-22

**Authors:** Qian Ji, Shengqiao Fu, Hao Zuo, Yumeng Huang, Liangmei Chu, Yanyan Zhu, Jing Hu, Yuting Wu, Shuangwei Chen, Yue Wang, Yongfei Ren, Xi Pu, Na Liu, Rongkun Li, Xu Wang, Chunhua Dai

**Affiliations:** 1 Department of Radiation Oncology, Institute of Oncology, Affiliated Hospital, Jiangsu University, Zhenjiang, Jiangsu Province China; 2 Jiangsu Key Laboratory of Medical Science and Laboratory Medicine, School of Medicine, Jiangsu University, Zhenjiang, Jiangsu Province China; 3 Department of Burn and Plastic Surgery, Affiliated Hospital, Jiangsu University, Zhenjiang, Jiangsu Province China; 4 Department of Gastroenterology, Affiliated Hospital, Jiangsu University, Zhenjiang, Jiangsu Province China; 5 Department of Emergency Surgery, Affiliated Hospital, Jiangsu University, Zhenjiang, Jiangsu Province China; 6 Department of Neurology, The Affiliated People’s Hospital of Jiangsu University, Zhenjiang, Jiangsu Province China

**Keywords:** Molecular biology, Preclinical research

## Abstract

Lipid peroxidation-induced ferroptosis is a newly recognized type of programmed cell death. With the method of RNA sequencing, we found that irradiation (IR) markedly increased the expression of ferroptosis promotive genes, whereas reduced the expression of ferroptosis suppressive genes in murine intestine tissues, when compared with those of liver and lung tissues. By using ferroptosis inducer RSL-3 and inhibitor liproxstatin-1, we found that ferroptosis is essential for IR-induced intestinal injury. Acyl-CoA Synthetase Long-Chain Family Member 4 (ACSL4) is an important component for ferroptosis execution, and we found that ACSL4 expression was significantly upregulated in irradiated intestine tissues, but not in liver or lung tissues. Antibacterial and antifungal regents reduced the expression of ASCL4 and protected against tissue injury in irradiated intestine tissues. Further studies showed that troglitazone, a ACSL4 inhibitor, succeeded to suppresses intestine lipid peroxidation and tissue damage after IR.

## Introduction

Radiation enteritis is an intestinal complication caused by IR for pelvic, abdominal, and retroperitoneal malignant tumors [1]. Abdominal pain, diarrhea, mucous bloody stools, and even intestinal obstruction and intestinal perforation may occur in these patients who have undergone abdominal radiotherapy [2, 3]. At present, there is no effective drug for radiation enteritis, which still needs further investigations [4]. There are 10 trillion microorganisms in the human intestinal tract, which can affect body weight and digestive ability, resist the risk of infection and autoimmune diseases, and control the response of the human body to cancer treatment drugs [5, 6]. Previous studies have indicated the essential role of enteric microorganisms in intestinal inflammatory and tumorous diseases [7–9]. Targeting intestinal microorganisms may provide novel strategies for radiation enteritis treatment [10].

With the emergence of ferroptosis, a newly discovered regulatory cell death form, more and more studies have shown that tissue damage after radiotherapy is closely related to ferroptosis [11, 12]. Ferroptosis is a form of iron-dependent regulatory cell death, which is caused by excessive lipid peroxidation [13–15]. Compared with other classical methods of cell death, the landmark feature is that a large number of iron ions and lipid-reactive oxygen species accumulate during cell death [16, 17]. Under the catalysis of iron ions in cells, the metabolic abnormality occurs, antioxidant capacity is weakened, lipid reactive oxygen species is accumulated, and intracellular oxidation-reduction is unbalanced, which eventually leads to cell death [18, 19]. Ferroptosis plays an important role in the occurrence and development of many diseases, including acute kidney injury, ischemia-reperfusion injury, Parkinson’s disease, and radiation resistance [15, 20–22]. Cells can secrete factors that activate the immune system through ferroptosis, and regulate cell inflammation, signal transduction, and cell growth [23]. Excessive inflammation will also lead to damage to the body [24].

The main mechanism of ferroptosis is that, under the action of bivalent iron or ester oxidase, it catalyzes the highly expressed unsaturated fatty acids on the cell membrane and causes lipid peroxidation, thus inducing cell death [25, 26]. Acyl-CoA Synthetase Long-Chain Family Member 4 (ACSL4), a member of acyl-coenzyme a synthetase long-chain family, is a key enzyme for regulating lipid composition [27–29]. In recent years, it has been found that it can promote the esterification of arachidonic acid to phosphatidylethanolamine (PE), thereby activating polyunsaturated fatty acids (PUFAs) and affecting the transmembrane characteristics of PUFAs [29, 30]. Studies have shown that PUFAs are substrates of synthetic lipid signal transduction, and they must be esterified and oxidized to transmit ferroptosis signals [26, 31]. Therefore, ASCL4 is an important target for regulating ferroptosis [32, 33].

In this study, we found that radiation-induced ferroptosis could contribute to intestinal injury. Intestinal bacteria and fungi are essential for inducing ferroptosis, and the mechanism might be associated with the elevated expression of ASCL4.

## Results

### The effect of IR on transcriptome of the liver, lung, and intestine of mice

**Fig. 1:**
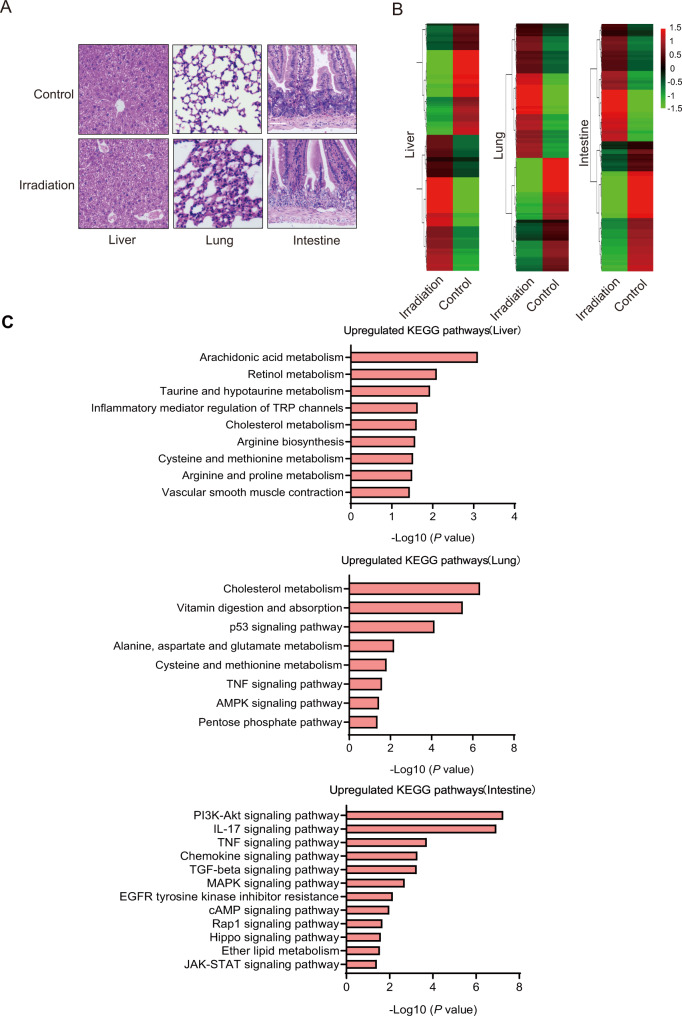
The effect of IR on transcriptome of liver, lung, and intestine of mice. **A** Representative images of the hematoxylin and eosin (H&E) staining of the liver, lung, and intestine from control and irradiated (IR) mice. The scale bar is 50 μm. **B** Changes of RNA transcription profiles in the liver, lung, and intestine tissues of the mice in the control group and IR group. Green bars represent down-regulation of gene expression and red bars represent up-regulation of gene expression. **C** KEGG Biological Process analyses differentially expressed genes in the liver, lung, and intestine of mice.

Hematoxylin-eosin (H&E) staining was performed to observe the morphological changes in lung, liver, and intestinal tissues after irradiation (IR). Compared with the control group, irradiated mice suffered significant tissue injuries, as lungs indicated thickening of the alveolar septum and a large amount of inflammatory cell infiltration, livers indicated necrosis and ballooning degeneration of hepatocytes, and intestines indicated the disarranged intestinal crypt and mucosal edema (Fig. 1A).

To systematically investigate the potential mechanisms that contribute to IR-induced acute tissue injuries, RNA sequencing of liver, lung, and intestines tissues was performed to compare the transcriptome alternations between control and irradiated mice. Results showed that there were significant differences in the transcription profiles of the liver, lung, and intestine (Fig. 1B). In liver tissue, 511 genes were significantly changed, with 268 genes upregulated and 243 genes downregulated; in lung tissue, 582 genes were significantly changed, with 218 genes upregulated and 364 genes downregulated; in intestinal tissue, 982 genes were significantly changed, with 704 genes upregulated and 278 genes downregulated (Supplementary Fig. 1). In order to further determine the specific molecular events that may be involved in tissues after IR, KEGG pathway analysis was performed. In irradiated lungs, pathways including p53 signaling pathway, Alanine, aspartate, glutamate metabolism, and TNF signaling pathway were enriched (Fig. 1C). In irradiated livers, pathways including Arachidonic acid metabolism, Inflammatory mediator regulation of TRP channels, and Cysteine and methionine metabolism were enriched. In irradiated intestines, pathways including the IL-17-signaling pathway, TNF signaling pathway, and Chemokine signaling pathway were enriched.

### Irradiation induces ferroptosis in intestinal tissue

**Fig. 2:**
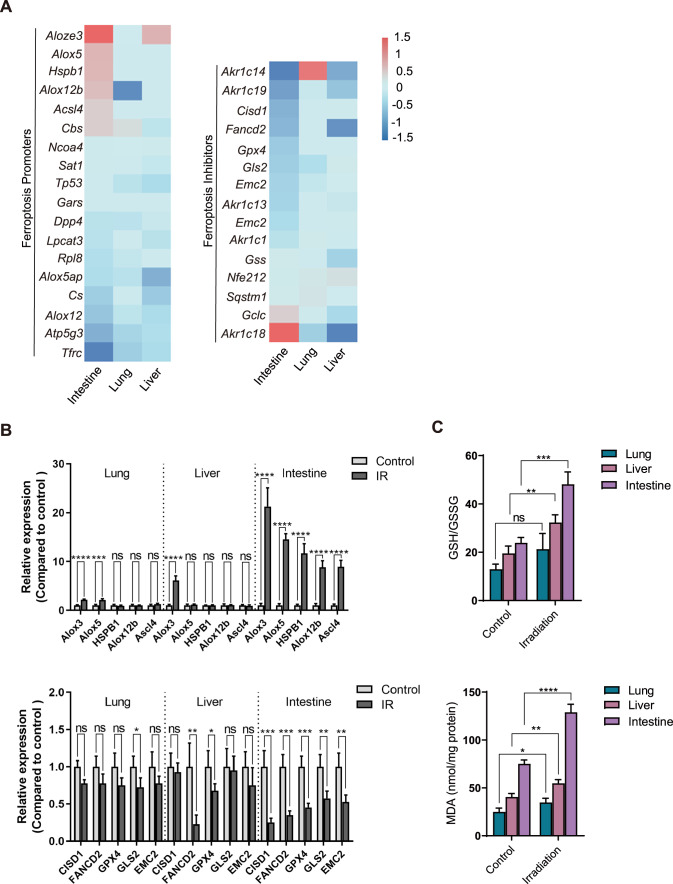
Irradiation induces ferroptosis in intestinal tissue. **A** Heatmap of ferroptosis promoters and inhibitors in liver, lung, and intestine tissues of mice after IR. **B** Reverse transcription polymerase chain reaction of ferroptosis-related genes in liver, lung, and intestine of mice. **C** Analysis of GSH/GSSG and MDA in liver, lung, and intestine after IR of mice. *P* values are derived from the permutation test, two-sided Student’s *t* test. *p < 0.05, **p < 0.01, ***p < 0.001, ****p < 0.0001.

Ferroptosis is a unique cell death mode driven by iron-dependent lipid peroxidation [15]. To assess whether ferroptosis was associated with IR-induced tissue injury, genes that promote or inhibit the ferroptosis process were analyzed. Compared with lung tissues and liver tissues, we noticed that expression of ferroptosis-promoting genes was apparently increased, whereas expression of ferroptosis-inhibiting genes was apparently reduced in intestine tissues (Fig. 2A). Further RT-qPCR studies confirmed the results (Fig. 2B and Supplementary Fig. 2). It suggests that ferroptosis may have potential roles in IR-induced intestine injury.

Reduced glutathione (GSH)/oxidized glutathione (GSSG) ratio is the main dynamic index of the redox state of cells. MDA is the end product of lipid oxidation [34, 35]. By measuring GSH/GSSG and MDA, ferroptosis was assessed in the lungs, livers, and intestines after IR. We noticed that compared with irradiated lungs and livers, GSH/GSSG ratio and MDA were markedly upregulated in intestines (Fig. 2C). Taking the alternations of ferroptosis-associated gene expression (Fig. 2A, B) into consideration, we suggest that ferroptosis is evident in irradiated intestine tissues, and which may contribute to IR-induced intestine injury.

### Ferroptosis promotes radiation injury in mice

**Fig. 3:**
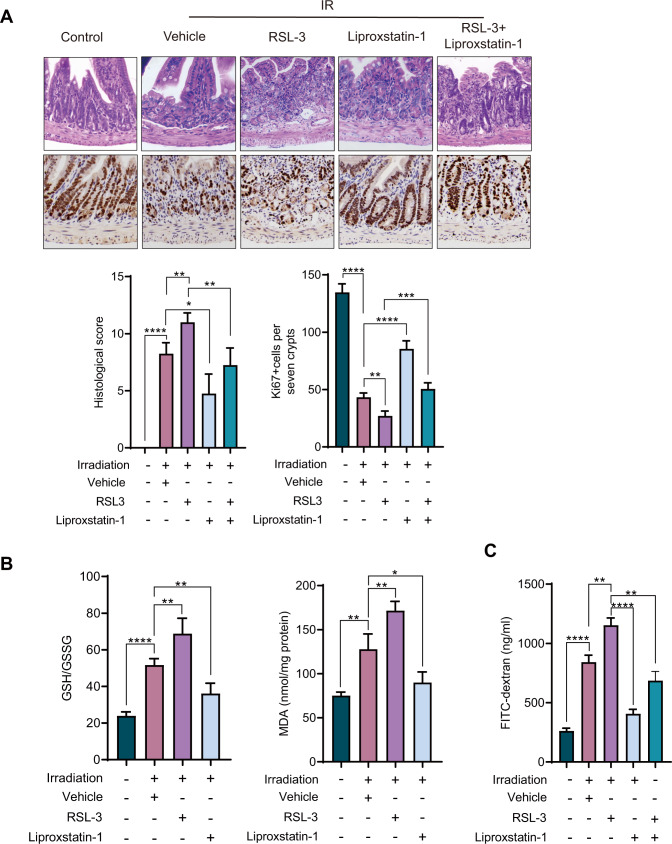
Ferroptosis promotes radiation injury in mice. **A** Histological scores of intestinal tissues of mice after IR. Ki67+ cells were counted in seven crypts. Representative images of the H&E staining and Ki67 staining of the intestine from control, vehicle, RSL-3, Liproxstatin-1, and RSL-3+Liproxstatin-1 groups. **B** Analysis of GSH/GSSG and MDA in intestinal control, vehicle, RSL-3, Liproxstatin-1, and RSL-3+Liproxstatin-1 of mice. **C** Analysis of intestinal permeability of mice in control, vehicle, RSL-3, liproxstatin-1, and RSL-3+Liproxstatin-1 treatment groups to FITC-Dextran. *P* values are derived from permutation test, two-sided Student’s *t* test. *p < 0.05, **p < 0.01, ***p < 0.001, ****p < 0.0001.

To determine whether ferroptosis contributes to radiation-induced intestine injury in mice, ferroptosis agonist RSL-3, inhibitor liproxstatin-1, and RSL-3+liproxstatin-1 were administrated to mice [36]. H&E staining showed that compared with the vehicle group (IR only), the intestinal tissues in RSL-3 group were obviously damaged. On the contrary, the tissue damage in liproxstatin-1 group was significantly reduced. The tissue damage score of RSL-3+liproxstatin-1 was lower than RSL-3 group and higher than liproxstatin-1 group. To further verify this result, we analyzed the cell viability of these tissues by staining Ki67. Compared with the vehicle group, there was a significant reduction of Ki67+ cells in RSL-3 group. However, the number of Ki67+ cells of liproxstatin-1 group was significantly higher than RSL-3 group and the RSL-3+liproxstatin-1 group was also higher than RSL-3 group (Fig. 3A). At the same time, we measured the MDA and GSH/GSSH radio. Compared with the vehicle group, the results showed that MDA and GSH/GSSH radio in RSL-3 group increased significantly, while MDA and GSH/GSSH radio in liproxstatin-1 group decreased significantly. The MDA and GSH/GSSH radio in RSL-3+liproxstatin-1 group was lower than RSL-3 group and higher than liproxstatin-1 groups (Fig. 3B). In addition, intestine permeability analyses of these groups were analyzed. Compared with the vehicle group, intestine permeability in mice treated with RSL-3 was significantly higher. However, in the mice treated with RSL-3+iproxstatin-1, intestine permeability was lower than RSL-3 group, and liproxstatin-1 group has the lowest intestine permeability (Fig. 3C). These results indicate that ferroptosis agonist promotes intestinal injury, while ferroptosis inhibitor reverses intestinal injury.

### Intestinal bacteria and fungi promote radiation intestinal injury and ferroptosis

**Fig. 4:**
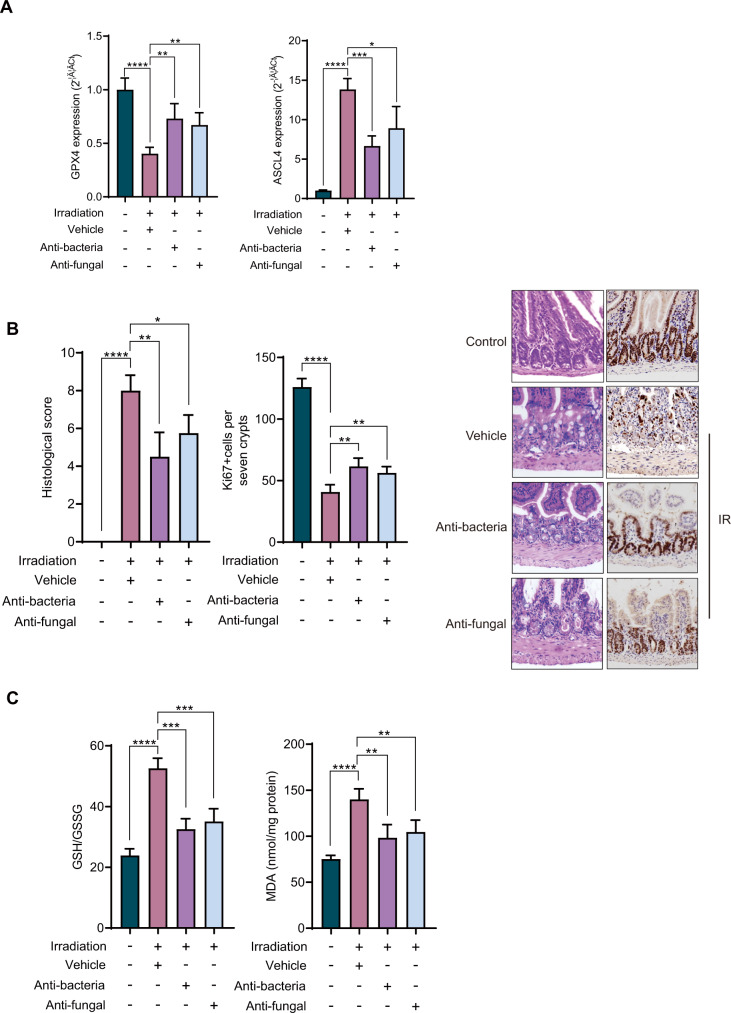
Intestinal bacteria and fungi promote radiation intestinal injury and ferroptosis. **A** Analysis of the expression levels of ferroptosis-related genes GPX4 and ACSL4 in intestinal tissues of mice in control, vehicle, antibacterial and antifungal. **B** Histological scores of intestinal tissues of mice after IR. Ki67+ cells were counted in seven crypts. Representative images of the H&E staining and Ki67 staining of the intestine from control, vehicle, antibacterial and antifungal. **C** Analysis of GSH/GSSG and MDA in intestinal control, vehicle, antibacterial, and antifungal of mice. *P* values are derived from permutation test, two-sided Student’s *t* test. *p < 0.05, **p < 0.01, ***p < 0.001, ****p < 0.0001.

Compared with liver and lung tissues, the expression of genes related to ferroptosis was apparent in intestine tissues. Therefore, we assume whether intestinal flora will play a certain role in it. In order to explore the influence of intestinal flora on radiation-induced intestinal injury and ferroptosis, we fed mice in an antibiotic group with antibiotic water for 2 weeks, and mice in the antifungal group were intraperitoneally injected with fluconazole for 2 weeks. After IR, we found that compared with the vehicle group, the expression of ferroptosis-related genes in intestinal tissues of mice in antibacterial group and antifungal group changed significantly. The expression of genes that promoted ferroptosis such as GPX4 increased, while the expression of genes such as ACSL4 decreased (Fig. 4A). H&E staining showed that compared with the vehicle group, the damage to small intestine in antibacterial group was obviously reduced and that in antifungal group was also reduced. Ki67 immunostaining showed that compared with the vehicle group, there was a significant increase of Ki67+ cells in antifungal group and antibacterial group (Fig. 4B). In order to further clarify the relationship between intestinal flora and radiation-related ferroptosis, we also measured MDA and GSH/GSSH radio. The results showed that MDA and GSH/GSSH radio in the treatment group were significantly lower than those in the vehicle group (Fig. 4C). These results suggested that intestinal bacteria and fungi may aggravate ferroptosis of intestinal tissue after IR.

### Intestinal bacteria and fungi promote ferroptosis by inducing ACSL4 expression

**Fig. 5:**
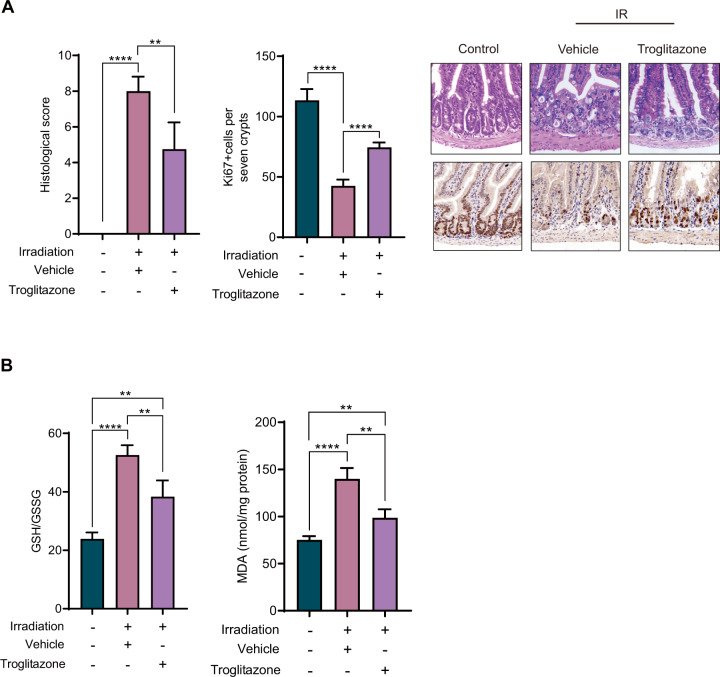
Intestinal bacteria and fungi promote ferroptosis by inducing ACSL4 expression. **A** Histological scores of intestinal tissues of mice after IR. Ki67+ cells were counted in seven crypts. Representative images of the H&E staining and Ki67 staining of the intestine from control, vehicle, and troglitazone. **B** Analysis of GSH/GSSG and MDA in intestinal control, vehicle, and troglitazone of mice. *P* values are derived from permutation test, two-sided Student’s *t* test. *p < 0.05, **p < 0.01, ***p < 0.001, ****p < 0.0001.

RNA sequencing results indicated that ferroptosis-related gene ACSL4 was markedly upregulated in intestine tissues, but not in liver and lung tissues after IR. In addition, we noticed that antibiotic and antifungal agents could suppress the expression of ASCL4. It suggests the potential role of ASCL4 in radiation-induced intestine injury. To further explore the effect of ACSL4 on intestinal ferroptosis after IR, we used ACSL4 inhibitor troglitazone to inject mice intraperitoneally. H&E staining showed that compared with the vehicle group, the tissue damage in troglitazone group was alleviated. Immunohistochemical showed that compared with the vehicle group, the troglitazone treatment resulted in an increase in Ki67 staining. (Fig. 5A). At the same time, we measured MDA and GSH/GSSH radio, which showed that compared with the vehicle group, the MDA and GSH/GSSH radio in troglitazone group decreased significantly (Fig. 5B). These results suggest that intestinal bacteria and fungi-induced ACSL4 expression may be associated with the ferroptosis and subsequent intestinal injury after IR. To further verify the protective effect of troglitazone on radiation-induced intestine injury, the observation periods were extended to 7 and 14 days. We found that radiation-induced intestine injury was the most evident at 3 days, and at 7 and 14 days, the tissue injury was gradually recovered (Supplementary Fig. 3). Similar results were obtained when detecting the MDA content. Troglitazone significantly alleviates tissue injury and lipid peroxidation at 3 and 7 days, and at 14 days the intestinal tissues were almost as normal. These results indicate that inhibition of ferroptosis with troglitazone shows a continuous protective effect on intestinal tissue and accelerates the recovery after radiation.

## Discussion

The gastrointestinal system is sensitive to radiation, and the intestinal injuries caused by IR, such as diarrhea, bleeding, and perforation, often affect the therapeutic effect [2, 3, 37]. For the long-term survival of cancer patients, it is necessary to relieve the radiation toxicity of the intestinal tract [38, 39]. In order to find potential intervention targets, RNA sequencing was performed in the liver, lung, and intestine tissues of mice after radiotherapy. We found that the expression of ferroptosis-related genes were obviously altered in intestinal tissues. Using ferroptosis inducer and inhibitor, we verified that ferroptosis was essential for IR-induced intestine injury.

Distinct from the liver and lung, intestine is filled with intestinal bacteria and fungi [40, 41]. Accumulating reports have confirmed that intestinal bacteria and fungi are required for regulating the process of inflammation and tumors in the digestive system [9, 42]. We suppose that bacteria and fungi in intestine may account for the ferroptosis after IR. To address our speculation, we set up mice in the vehicle group, antibacterial group, and antifungal group for comparison. The results showed that the intestinal tissue damage and lipid peroxidation of mice in antibacterial treatment group were significantly lower than those in the vehicle group.

ACSL4 is a key enzyme regulating lipid composition, which can induce ferroptosis by activating iron oxidation of PUFAs [36]. We found that IR could significantly increase ACSL4 expression in intestine tissues but not in lung and liver tissues, which was then reserved by antibacterial and antifungal regents. Until now, commercial ACSL4-specific inhibitors cannot be obtained. Troglitazone, an antihyperglycemic agent, has been reported as a potent inhibitor of ACSL4 [43, 44]. In this study, we found that inhibition of ACSL4 with troglitazone inhibits lipid peroxidation of intestinal tissues and protects against tissue injury. It suggests that ACSL4 may be a potential target for treating radiation enteritis. Further investigations for the discovery of specific ACSL4 inhibitors may facilitate the therapy of radiation enteritis.

## Materials and methods

### Animal models

All animal experiments were conducted according to the National Institutes of Health’s Guidelines for the Care and Use of Laboratory Animals. We used the C57BL/6 female mice, aged 6–8 weeks, weighing about 25 g, to get food and water freely. Mice in RSL-3 group were intraperitoneally injected with 10 mg/kg daily, two days before IR. Lipostain-1 mice were intraperitoneally injected with 2.5 mg/kg daily, two days before IR. Mice in IR group were injected intraperitoneally with the vehicle daily. The mice in the antibacterial group were continuously fed with antibiotic water (metronidazole 1 mg/ml, vancomycin 0.5 mg/ml, streptomycin 0.5 mg/ml, ampicillin 0.5 mg/ml) for 2 weeks. Mice in the antifungal group were intraperitoneally injected with fluconazole (2 mg/ml) for 2 weeks. Mice in troglitazone group were intraperitoneally injected with troglitazone(10 mg/ml) 12 hour before IR. After being executed humanely, the liver, lung, and intestine tissues of mice were taken for ferroptosis-related indicators and tissue damage assessment.

### IR

The mice were anesthetized with ether and fixed on the foam board, and the whole body was exposed to radiation. IR with Varian 23EX linear accelerator device, the IR dose is 14 Gy and the dose rate is 400 Mu/min.

### RNA sequencing

According to the manufacturer’s instructions, we used TRLzol reagent to separate the total RNA from the RNA-seq of the liver, lung, and intestine tissues of mice after radiotherapy, and sent it to Novogene Co for detection, so as to obtain the related RNA sequencing. According to the manufacturer’s instructions, we used TRLzol reagent to separate the total RNA from the RNA-seq of liver, lung, and intestine tissues of mice after radiotherapy, and sent it to Novogene Co for detection, so as to obtain the related RNA sequencing data.ng data.

### H&E staining

Liver, lung, and intestine of mice were fixed with 10% formalin and embedded in paraffin. H&E staining method was used for staining, and then the tissues were observed under a dehydrated sealing microscope. The sections were stained with hematoxylin and eosin, and evidence of intestinal mucosal injury was quantified (0 = none, 1 = mild, 2 = moderate, 3 = high). The histological severity of radiation-induced intestinal damage was assessed by the degree of epithelial architecture maintenance, crypt damage, vascular dilation, and infiltration of inflammatory cells in the lamina propria.

### Immunohistochemistry

Mice were killed 3 days after IR, and the intestine was fixed with 10% formalin and then embedded in paraffin. Antigen retrieval was performed according to standard procedures. After antigen retrieval, the sections were incubated with serum at room temperature for 1 hour and then with anti-Ki67 antibody(1:300 dilution) overnight at 4 °C. After that, the sections were incubated in secondary antibody at 37 °C for 30 min. Finally, through IPP software, the image was obtained by the blind complement method, and then the positive staining was quantified objectively.

### Determination of serum FITC-dextran concentration

Mice fasted for 4 h and then FITC-Dextran (4 kDa, 600 mg/kg) was administered by gavage. After 4 h, blood was collected. The collected blood was diluted with physiological saline in equal volume, and placed at 4 °C in the dark for 4 h to produce coagulation. Blood was centrifuged (1200 rpm, 2 min) and the supernatant was collected and plated into 96-well plate, and then use fluorescent microplate instrument to measure the serum concentration of FITC-Dextran.

### RT-qPCR

UseTrizol reagent (Takara) and PrimeScript RT-PCR kit (Takara) to extract total RNA and reverse transcription RNA. Real-time PCR was performed using SYBR Premix Ex Taq to analyze gene expression at the recommended thermal settings. Each gene was normalized to β-actin mRNA levels, and the 2^(−ΔΔCt)^ method was used to calculate relative mRNA expression.

### MDA

MDA content in intestinal tissue of mice was determined by using Lipid Peroxidation (MDA) kit(ab118970) to monitor lipid peroxidation. About 10 mg of intestinal tissue was homogenized in 303 lysis solution (MDA lysis buffer + BHT) with a Dounce homogenizer sitting on ice, with 10–15 passes. Intestinal tissue homogenates were centrifuged at 13,000 × *g* for 10 min to remove insoluble material. Add 600 µL of TBA reagent into each vial containing 200 µL standard and 200 µL sample, incubate at 95 °C for 60 min, and then cool to room temperature in an ice bath for 10 min. Take 200 µL of the reaction mix and immediately add it into a 96-well microplate in the condition of OD532 nm for colorimetric analysis.

### GSH/GSSG

GSH/GSSG radio in intestinal tissue of mice was determined by using GSH/GSSG Radio Detection Assay kit(ab138881) to determine the cell redox state. About 20 mg of intestinal tissue was resuspended in 400 µL of ice-cold Mammalian Lysis Buffer and then homogenize e tissue with a Dounce homogenizer with 10–15 passes. Intestinal tissue homogenates were centrifuged at 13,000 g for 10 min to remove insoluble material. Finally, the supernatant was measured to calculate GSH/ GSSG ratio.

## Supplementary information

Supplemental Material

## Data Availability

The raw data of this article will be made available by the authors, without undue reservation.
