# Supplementary material for: ACSL4 is essential for radiation-induced intestinal injury by initiating ferroptosis
Source: Cell Death Discov. 2022 Jul 22;8:332. doi: 10.1038/s41420-022-01127-w (PMC9307849; doi:10.1038/s41420-022-01127-w)
Supplement: Supplementary file 1 — Supplemental Material [file 41420_2022_1127_MOESM1_ESM.docx]

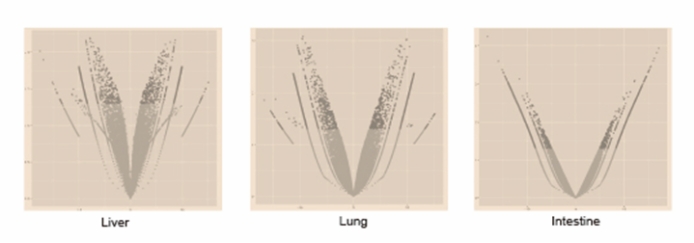


**Supplementary Figure 1:**

**The effect of IR on transcriptome of liver, lung and intestine of mice.**

Volcano plots of differential gene expression in liver, lung and intestine tissues of mice in control group and IR group. Light grey dots represent upregulated ferroptosis-related genes, and dark grey dots represent downregulated ferroptosis-related genes.


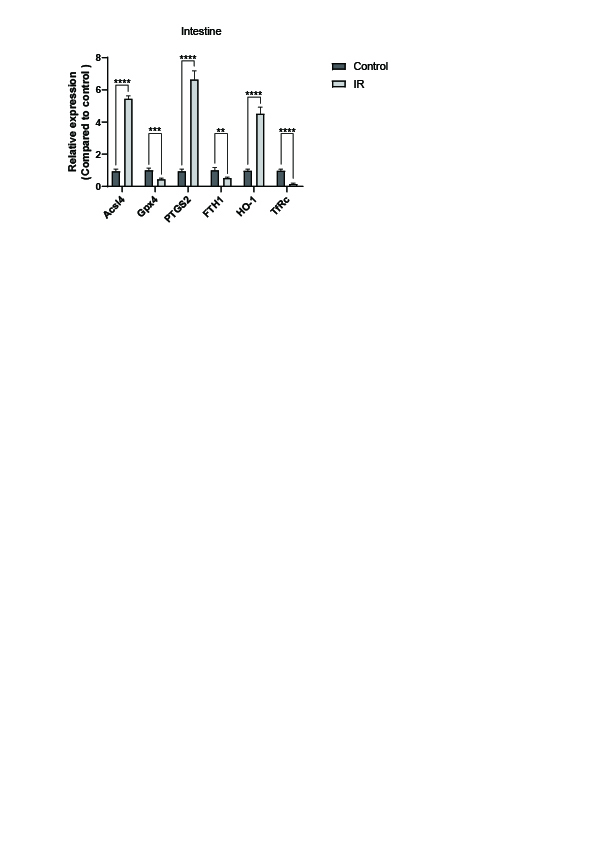


**Supplementary Figure 2:**

**Irradiation induces ferroptosis in intestinal tissue.**

Reverse transcription polymerase chain reaction of ferroptosis-related genes in intestine of mice.


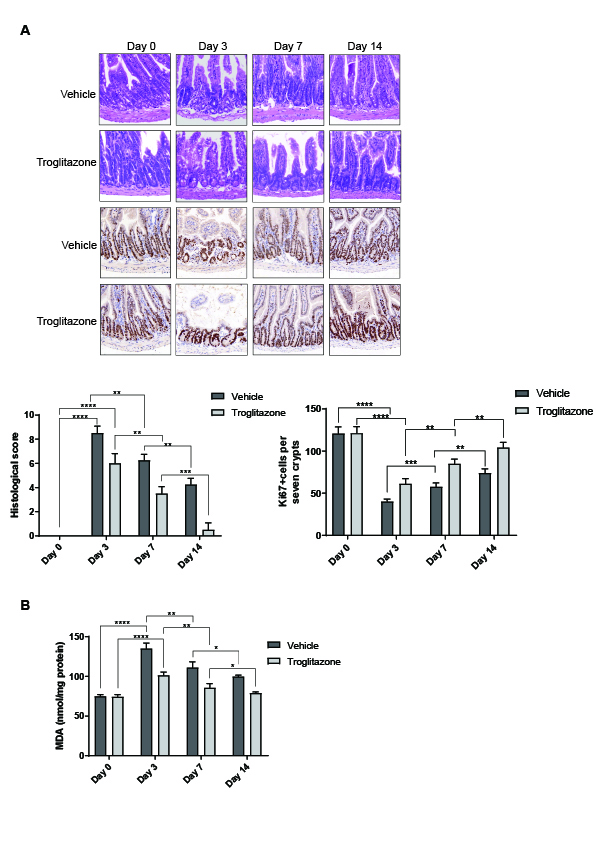


**Supplementary Figure 3:**

**Intestinal bacteria and fungi promote ferroptosis by inducing ACSL4 expression.**

**A** Histological scores of intestinal tissues of mice after IR in vehicle and troglitazone after H&E staining. Ki67+ cells were counted in seven crypts. Representative images of the H&E staining and Ki67 staining of the intestine from vehicle and troglitazone at day 0,day 3,day 7 and day 14. **B** Analysis of MDA in intestinal vehicle and troglitazone of mice. *P* values are derived from permutation test, two-sided Student’s t test. ^*^*p*<0.05, ^**^*p*<0.01, ^***^*p*<0.001, ^****^*p*<0.0001.
